# Therapeutic hypothermia after cardiac arrest during living-donor liver transplant surgery: A case report

**DOI:** 10.1097/MD.0000000000022513

**Published:** 2020-11-06

**Authors:** Jaesik Park, Ju Eun Kwak, Yun Jeong Cho, Ho Joong Choi, Hoon Choi, Min Suk Chae, Chul Soo Park, Jong Ho Choi, Sang Hyun Hong

**Affiliations:** a Department of Anesthesiology and Pain Medicine; b Department of Surgery, Seoul St. Mary's Hospital, College of Medicine, The Catholic University of Korea, Seoul, Republic of Korea.

**Keywords:** cardiac arrest, therapeutic hypothermia, liver regeneration, living-donor liver transplantation

## Abstract

**Rationale:**

Therapeutic hypothermia is an effective medical treatment for neurological recovery after cardiac arrest. Here, we describe a case of successful mild therapeutic hypothermia after cardiac arrest during living-donor liver transplantation.

**Patient concerns:**

A 54-year-old woman with alcoholic liver cirrhosis was admitted for living-donor liver transplantation. Cardiac arrest occurred during the anhepatic phase. After cardiopulmonary resuscitation, spontaneous circulation returned, but the bispectral index level remained below 10 until the end of surgery.

**Diagnoses:**

Neurological injury caused by global cerebral hypoperfusion was suspected.

**Interventions:**

The patient was treated with mild therapeutic hypothermia for 24hours after resuscitation targeting a core body temperature of 34°C with surface cooling using ice bags.

**Outcomes:**

The patient recovered consciousness about 22 hours after the event. However, she showed symptoms of delirium even when discharged. At the 3-month follow-up exam, she showed no specific neurological complications. The transplanted liver showed no problems with regeneration.

**Lessons:**

Mild therapeutic hypothermia may be safely adopted in cases of cardiac arrest in liver transplant patients and is beneficial for neurological recovery.

## 1 Introduction

Therapeutic hypothermia is a proven medical treatment for neurological protection after cardiac arrest or traumatic brain injury. Numerous reports support the effectiveness of hypothermia in improving the neurological outcome after such events.^[1,2]^ Previous reports described the adoption of therapeutic hypothermia after intraoperative cardiac arrest.^[3]^ However, there are few reports of induced hypothermia in patients undergoing liver transplantation surgery. In this report, we present a case of a successfully resuscitated patient with mild therapeutic hypothermia after intraoperative pulseless electrical activity (PEA) during living-donor liver transplantation (LDLT).

## 2 Case report

A 54-year-old woman (height, 157 cm; weight, 62 kg; blood type, A+) with alcoholic liver cirrhosis (model for end-stage liver disease score, 20) was admitted for LDLT. She had been taking an endothelin receptor antagonist (bosentan) for pulmonary hypertension and a beta blocker (carvedilol) for atrial fibrillation. A transthoracic echocardiogram showed left atrium (LA) enlargement (diameter, 43 mm; volume, 73 ml), mild aortic regurgitation, and mild tricuspid regurgitation with moderate pulmonary hypertension (right ventricular systolic pressure, 61 mmHg). The contractile function of the left ventricle (LV) was within the normal range (ejection fraction, 63.3%; s’, 7.1 cm/s) without segmental wall motion abnormalities. However, moderate diastolic dysfunction of the LV was noted (average E/e’, 7.7; E/A, 0.9; septal e’, 6.2 cm/s; tricuspid regurgitation velocity, 3.6 m/s; left atrium velocity index; 46.5 ml/m^2^). Electrocardiography revealed a prolonged QTc interval (504 s).

LDLT using the donor's right hepatic lobe was performed using the piggyback technique. The donor liver graft was from her daughter, a 33-year-old woman (blood type, A+), and showed little fatty change. The initial mean pulmonary arterial pressure (PAP) of 24 mmHg checked after pulmonary arterial catheter insertion and central venous pressure (CVP) was 10 mmHg. The cardiac index (CI), stroke volume variation (SVV), and systemic vascular resistance index (SVRI) were within the respective normal ranges. During the early preanhepatic stage, mild systolic hypotension of 75 to 85 mmHg was observed. After bolus injections of ephedrine and phenylephrine, a norepinephrine infusion was begun at a dose of 0.15 to 0.25 mcg/kg/min to maintain blood pressure. In the late preanhepatic stage when the estimated blood loss exceeded 1000 ml, we began blood product transfusion with a rapid infusion system.

**Figure 1:**
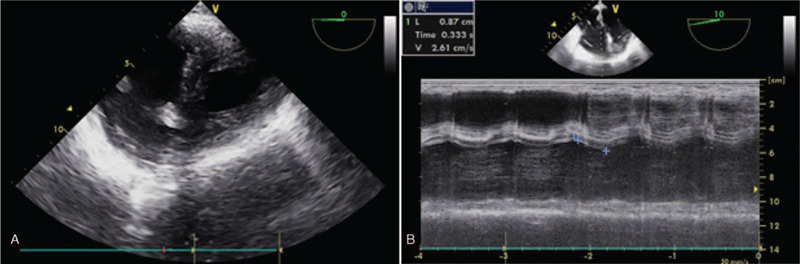
Transesophageal echocardiography findings immediately after the return of spontaneous circulation. In the transgastric mid-papillary short-axis view (A), the right ventricle was seen to be larger than the left ventricle in diastole. M-mode in the mid-esophageal four-chamber view (B) showed a decreased tricuspid annular plane systolic excursion of 8.7 mm (normal range, 15–20 mm).

About 30 minutes after the start of the anhepatic stage, a sudden drop in systemic blood pressure coupled with a decreased heart rate were observed (Table 1). At the same time, the PAP and CVP increased to more than 60 mmHg and 30 mmHg, respectively. We immediately injected glycopyrrolate and nitroglycerin and began infusing dobutamine at a rate of 6 mcg/kg/min, and we increased the infusion rate of norepinephrine. However, the patient's systolic blood pressure and heart rate decreased to less than 30 mmHg and 35 beats/min, respectively. The surgeon was promptly asked to perform cardiac massage after opening the diaphragm, and boluses of epinephrine were administered. The patient recovered circulation within about 10 minutes. As soon as the patient recovered circulation, we drew 600 ml of blood through the central venous catheter due to suspected right heart failure and examined the heart by transesophageal echocardiography (TEE). TEE showed the right atrium (RA) and right ventricle (RV) to be enlarged in both diastole and systole (Fig. 1). Tricuspid annular plane systolic excursion (TAPSE) was measured as 8.7 mm (normal range, 15–20 mm) and the maximum tricuspid regurgitation velocity (TR Vmax) was 124 cm/s (normal range, ≤ 280 cm/s). The ejection fraction of the LV was about 60%. The blood pressure and heart rate of the patient increased after the recovery of circulation. At the same time, the PAP and CVP decreased. The operation was resumed and the patient's vital signs remained stable with a mean blood pressure of 65 mmHg or higher. However, there was a substantial drop in the bispectral index (BIS). The BIS level, which remained within the range of 40 to 45 before cardiac arrest, decreased to 0 to 10 (Table 1). In addition, the pupils were fixed in dilation (8 mm) and did not respond to light stimuli. We discussed the situation with the surgeon and decided to induce hypothermia during surgery. We attempted to keep the body temperature at about 34 °C using ice bags.

**Table 1:** Hemodynamic parameters, bispectral indices and coagulation profiles during the perioperative period.

Perioperative stage	Preanhepatic	Anhepatic							Neohepatic		Postoperative					
				
Perioperative event	Beginning of surgery	Portal vein clamping	Cardiovascular collapse						Portal vein declamping	Peritoneal closure	Intensive care unit admission					
						
Time points			Every 5 min after cardiac arrest (CA)						CA + 1 h	CA + 3 h	CA + 4 h	CA + 8 h	CA + 12 h	CA + 16 h	CA + 24 h	CA + 34 h
Hemodynamic parameters, bispectral indices and coagulation profiles																
Systolic arterial pressure (mmHg)	124	128	67	54	39	29	19	95	74	100	135	117	123	139	120	105
Diastolic arterial pressure (mmHg)	65	75	40	33	33	20	15	52	47	55	81	72	64	77	80	48
Heart rate (beats/min)	68	61	45	41	40	35	25	70	84	75	76	87	70	61	57	81
–	–	–	–	–	–	–	–	–	–	–	–	–	–	–	–	7
–	–	–	–	–	–	–	–	–	–	–	–	–	–	–	–	–
–	–	–	–	–	–	–	–	–	–	–	–	–	–	–	–	–
SpO_2_ (%)	≥95	≥95	≥95	≥95	≥95	≥95	≥95	≥95	≥95	≥95	≥95	≥95	≥95	≥95	≥95	≥95
Cardiac Index (L/min/m^2^)	2.7	2.1	2.9	–	–	–	–	–	3.6	2.6	–	–	–	–	–	–
Systemic Vascular Resistance Index (dyn/s/cm ’/m”)	2622	2661	874	–	–	–	–	–	1640	1607	–	–	–	–	–	–
Bispectral index	43	41	40	–	–	–	–	–	5	0∼10	0∼5	–	–	–	–	–
Body temperature (°C)	35.3	35.2	34.6	34.2	34.1	34.1	34.1	34.1	34.1	34.3	34.1	34.7	34.1	34.4	34.1	35.9
INR	1.73	3.57	–	–	–	–	–	–	2.59	2.2	2.19	–	2.32	1.69	1.46	1.49
aPTT (seconds)	49	120	–	–	–	–	–	–	120	71.7	64.2	–	56.9	50.7	41.5	38.9
Platelet count	59k	29k	–	–	–	–	–	–	18k	20k	20k	–	21k	12k	82k	92k

The operation was terminated within 4 hours after arrest without recovery of the BIS level and dilation of the pupils. The patient was transferred to the intensive care unit (ICU) in an intubated state with a continuous infusion of norepinephrine, dobutamine, and vasopressin. The total operation time was 215 minutes and the estimated blood loss was 3000 ml. During the operation, 10 units of packed red blood cells and 9 units of fresh frozen plasma were administered along with 4500 ml of crystalloids and 500 ml of colloids.

On arrival at the ICU, the patient was comatose and both pupils were fixed at 8 mm. Spontaneous blinking and jerking of both limbs were observed 30 minutes after ICU admission. Therapeutic hypothermia was continued with ice bags and the room temperature of the ICU room was decreased to aid hypothermia. Intravenous cold saline administration for the induction of hypothermia was not adopted to avoid accelerating coagulopathy. Through this method, we maintained mild therapeutic hypothermia targeting a body core temperature of 34 °C. About 22 hours after the event, the patient's mental status improved and she was able to open her eyes spontaneously. She began to nod her head in response to simple questions. Therapeutic hypothermia was terminated at 24 hours after cardiac arrest.

The patient reached a normal body temperature 28 hours after the event. However, for the next 6 hours, more than 1000 ml of blood were drained and the patient's blood pressure dropped gradually. Emergency liver computed tomography (CT) angiography revealed a large hematoma in the abdomen. The patient underwent an emergency operation and re-suturing of the inferior vena cava clipping site was performed. No more internal bleeding was observed after the re-operation.

The patient was extubated on the third postoperative day after the first operation. On the 7th postoperative day, the patient had recovered sufficiently to start a liquid diet, but began to show thought disturbance, saying inappropriate words. A change in personality was also observed, with constant laughing and talking. Compared to the right lobe volume before transplantation, a CT scan showed that the volume of the transplanted liver was increased to 166% on the 7th postoperative day.

On the 13th postoperative day, the patient was transferred to the general ward. She showed impaired orientation regarding time and place and behaved as though she was in her 30s, but the ability to recognize people was maintained. Brain magnetic resonance imaging (MRI) showed no specific findings other than cerebral manifestations of chronic liver disease.

The patient was discharged on the 28th postoperative day. At the 3-month follow-up exam, the patient's delirium and other symptoms had improved and she showed no neurological complications.

Informed consent for publication was obtained from the patient and The Institutional Review Board of Seoul St. Mary's Hospital Ethics Committee approved the present study.

## 3 Discussion

Here, we described the successful application of mild therapeutic hypothermia after intraoperative cardiac arrest during liver transplant surgery. Although the patient underwent re-operation for postoperative bleeding, it appeared to be due to surgical problems and not to medical bleeding caused by hypothermia. The hypothermia therapy did not affect graft regeneration after transplantation.

Therapeutic hypothermia is usually indicated for neurological protection in patients with a stroke or traumatic brain injury. There have been cases of therapeutic hypothermia for neurosurgical procedures, post-cardiopulmonary resuscitation (CPR) or traumatic brain injuries, and the cases support the suggestion that therapeutic hypothermia could be helpful to improve neurological outcomes.^[2,3]^ However, there have been no previous reports regarding therapeutic hypothermia in cases of LDLT.

Contraindications for therapeutic hypothermia include uncontrollable hypotension and coagulopathy. ^[4]^ Hypotensive and coagulopathic patients were excluded from the Hypothermia after Cardiac Arrest Study Group and Bernard studies, respectively.^[1,2]^ These patients were excluded because of possible adverse effects of hypothermia on hemodynamic and coagulation functions. However, in other reports, therapeutic hypothermia was helpful in patients with cardiogenic shock after CPR.^[5,6]^ In addition, in one report mild therapeutic hypothermia did not negatively affect coagulopathy.^[7]^ In the present case, therapeutic hypothermia did not exacerbate coagulopathy after liver transplant surgery even though the patient underwent re-operation for intraabdominal bleeding due to surgical problems.

Mild therapeutic hypothermia is induced hypothermia with body core temperature of 32 °C to 34 °C or 35 °C.^[1,8,9]^ Two randomized clinical trials showed that mild therapeutic hypothermia after cardiac arrest is helpful for improving neurological outcomes. Bernard et al^[2]^ reported that the Glasgow Outcome Coma Scale score was better and mortality was lower in the hypothermia group than in the normothermia group. In addition, the Hypothermia after Cardiac Arrest Study Group reported that patients with hypothermia showed favorable neurological outcomes based on cerebral performance category and lower mortality.^[1]^

There are two ways to induce hypothermia: surface cooling and endovascular cooling. Surface cooling includes the use of exposure, water mattresses, cooling jackets, and ice bags.^[10,11]^ Surface cooling is slower, but can prevent complications related to the endovascular approach. Endovascular cooling requires a shorter time to reach the target temperature. However, complications such as cardiac arrhythmia and thrombocytopenia can occur.^[12]^ We used ice bags to avoid the possible complications related to the endovascular approach, and the temperature was maintained at 34 °C.

Right heart failure is associated with increased mortality and morbidity, especially perioperatively.^[13]^ Right heart failure could occur under conditions of increased afterload, decreased preload, and reduced right ventricular contractility, and can occur due to pulmonary embolism, right ventricular infarction, constrictive pericarditis.^[13–15]^ The assessment of right ventricular function is challenging because of the complex shape of the RV.^[15,16]^ Decreased TAPSE is associated with dysfunction of the RV and is correlated with an increased mortality rate.^[17]^ If the RV exceeds the size of the LV, RV dilatation can be assumed.^[18]^ In our case, as an enlarged RV and decreased TAPSE with pulmonary hypertension were observed, right ventricular dysfunction caused by an increased afterload was suspected. But abnormal wall motions in RV were not seen on TEE. PAP also indicated the presence of pulmonary arterial hypertension and right heart dysfunction. Pulmonary thromboembolism was not identified through TEE, but it was suspected.

Phlebotomy could be helpful to intervene in cases of right heart failure. Acute pulmonary arterial hypertension could induce right ventricular dysfunction and systemic hypotension.^[19]^ In our case, PAP and CVP were acutely elevated and intractable hypotension was maintained despite the use of vasopressors and inotropic agents, resulting in PEA. Therapeutic phlebotomy was performed immediately after CPR in our case. Eventually, the CVP and PAP decreased and the hemodynamic state became stable.

Graft regeneration after surgery is important for the prognosis of LDLT recipients. Our concern was that hypothermia would slow the metabolic rate of the graft liver and impede the process of liver regeneration. It has been reported previously that hypothermia may impair hepatic regeneration in acute liver failure.^[20]^ However, in another report, therapeutic hypothermia at 32 to 35 °C was shown to be helpful in cases of acute hepatic failure without complications, such as bleeding.^[8]^ We chose mild therapeutic hypothermia in the present case, and no, problems were encountered in graft regeneration. In a previous study, the volume of the grafted right lobe was about 165.8% at 1 week after surgery compared to the grafted liver volume at the time of surgery.^[21]^ In our case, the transplanted liver had grown to 166% by 1 week after surgery.

In summary, mild therapeutic hypothermia can be applied to improve neurological outcomes after perioperative cardiac arrest in liver transplant patients. In the present case, therapeutic hypothermia neither worsened coagulopathy nor slowed graft regeneration after liver transplant surgery. However, the efficacy and safety of therapeutic hypothermia after cardiac arrest during liver transplant surgery should be confirmed in future studies.

## Author contributions

**Conceptualization:** Sang Hyun Hong.

**Data curation:** Ju Eun Kwak, Yun Jeong Cho.

**Formal analysis:** Min Suk Chae, Ho Joong Choi.

**Investigation:** Yun Jeong Cho, Hoon Choi.

**Supervision:** Sang Hyun Hong, Chul Soo Park.

**Validation:** Sang Hyun Hong, Jong Ho Choi.

**Writing – original draft:** Jaesik Park, Ju Eun Kwak.

**Writing – review & editing:** Sang Hyun Hong, Jaesik Park.
